# Abdominal Ecchymosis: Emergency, or Urgen-C?

**DOI:** 10.7759/cureus.38091

**Published:** 2023-04-25

**Authors:** Riley Jones, Leila Shafiq, Benmichael Idowu, Nila S Radhakrishnan, Jason Fromm

**Affiliations:** 1 Medicine, University of Florida College of Medicine, Gainesville, USA; 2 Medicine, University of South Florida Morsani College of Medicine, Tampa, USA

**Keywords:** food insecurity, psychosocial factors, malnutrition, scurvy, vitamin c deficiency, chatgpt

## Abstract

Scurvy is a multisystem disease caused by vitamin C deficiency, historically associated with lethargy, gingivitis, ecchymosis, edema, and death if left untreated. Contemporary socioeconomic risk factors for scurvy include smoking, alcohol abuse, fad diets, mental health conditions, social isolation, and economic marginalization. Food insecurity is also a risk factor. This report describes a case of a man in his 70s who presented with unexplained dyspnea, abdominal pain, and abdominal ecchymosis. His plasma vitamin C level was undetectable, and he improved with vitamin C supplementation. This case highlights the significance of awareness of these risk factors and emphasizes the need for a comprehensive social and dietary history to enable the timely treatment of this rare but potentially fatal disease.

## Introduction

Scurvy is a disease caused by a deficiency in ascorbic acid, an essential dietary nutrient that plays a critical role in collagen synthesis. As a cofactor for proline and lysine hydroxylases, vitamin C (VC) stabilizes the tertiary structure of the collagen molecule and promotes its gene expression [1]. When VC stores are depleted, and serum concentrations fall below 0.2mg/dL (11uM/L), disequilibrium occurs between non-VC dependent tissue remodeling collagenases resulting in systemic degradation and friability of connective tissues. This underlying pathophysiologic process leads to the clinical manifestations of scurvy, which include lethargy, gingival hypertrophy and bleeding, easy bruising, edema of the extremities, and death if left untreated.

Although scurvy is most commonly associated with the vitamin C-deficient diets of seafaring explorers in the 15th and 16th centuries, descriptions of the disease date back to as early as 1550 BC Egypt [2-5]. The Scottish naval surgeon James Lind is credited with the first observation that eating lemons and oranges could produce a relatively quick cure [6]. Centuries later, scurvy is recognized as a multisystem disease resulting from VC deficiency in acute micronutrient malnutrition. However, it has a complex relationship with modern socioeconomic risk factors, including smoking, alcohol use disorder, fad diets, mental health conditions, social isolation, and low socioeconomic status [2,7]. Food insecurity is also a risk factor as it is directly related to a suboptimal diet and can be seen as a ramification of economic marginalization [7]. Intensifying regional conflicts have caused starvation. These famine conditions occur in areas with high soil fertility and highlight the sociopolitical dimensions of modern scurvy. 

## Case presentation

**Figure 1:**
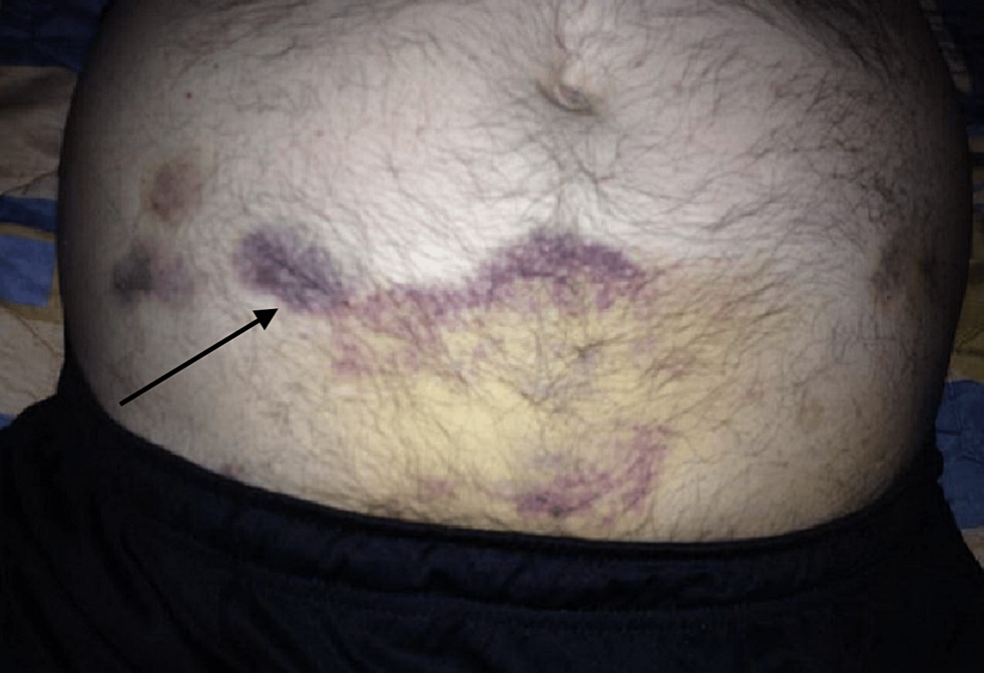
Ecchymosis

**Figure 2:**
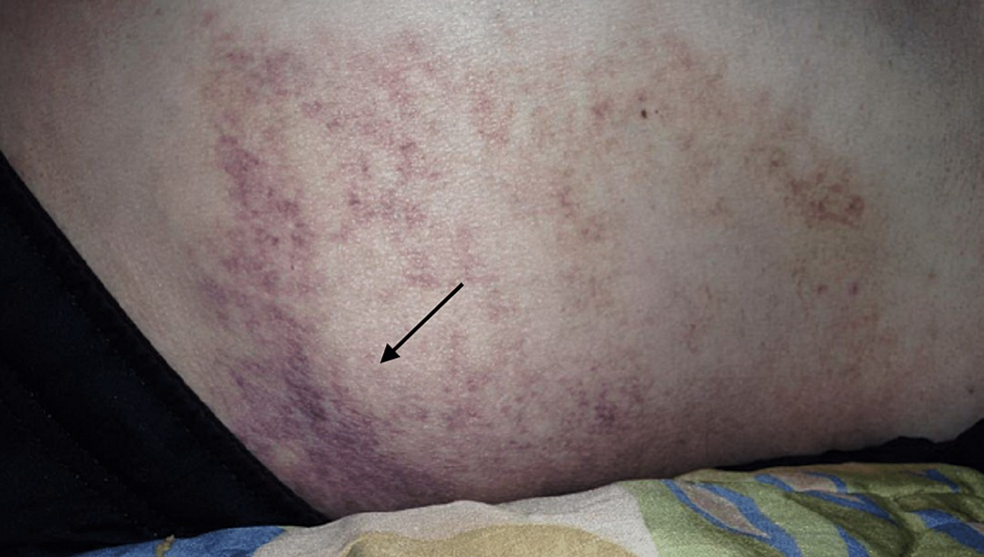
Flank Ecchymosis

A man in his 70s with a history of tobacco abuse, depression, and anxiety presented with complaints of dyspnea and abdominal pain. The patient had been experiencing progressively worsening dyspnea for the past three days, which he attributed to feeling anxiety. He also reported experiencing generalized abdominal pain and unexplained ecchymosis which was not associated with trauma and had an unknown time frame Figures 1, 2.

**Figure 3:**
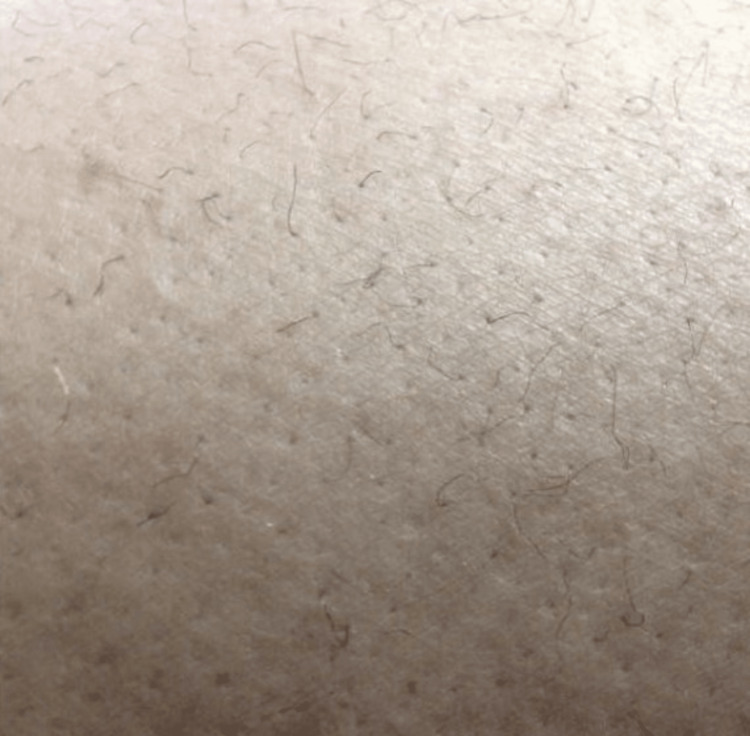
Corkscrew hair

The patient appeared mildly dehydrated and was tachypneic upon presentation. Examination revealed significant ecchymosis across his lower abdomen. The patient had missing teeth, which he had lost progressively over years, and changes in his lower extremity hairs that were later identified to be "corkscrew" in nature Figure 3.

Initial laboratory studies revealed a hemoglobin level of 10.9 g/dL (reference range 13.5-16.5 g/dL) and a mean corpuscular volume of 91.1 fL (reference range 80-100 fL). Workup for dyspnea revealed a normal alveolar-arterial gradient and no evidence of acidosis. The patient was initially diagnosed with an acute anxiety attack, and his dehydration was resolved with IV fluids. Further exploration of the unexplained abdominal ecchymosis revealed negative workup results for infection, renal failure, cirrhosis, and coagulopathy.

A more detailed psychosocial history revealed that the patient was living in isolation in an apartment and was unable to care for himself adequately due to agoraphobia and deep depression triggered by the loss of his mother. He had been prescribed escitalopram four years prior to admission. Six months prior to admission, his dose of escitalopram was increased from 10mg to 20mg. However, at the time of admission, he was not taking any medication. For the past several years, he had been eating canned foods and meats with no fresh fruits or vegetables. Given his exam findings in the presence of a vitamin C-devoid diet, scurvy was suspected and confirmed when a serum vitamin C level was below the level of detection. He was not screened for other micronutrient deficiencies since processed grains and meat contain chromium, copper, selenium, and zinc. 

Dietary and social work interventions were initiated, and the patient was prescribed supplemental vitamin C. Ascorbic acid 250mg was administered orally daily starting on the day of admission. His anxiety improved within 36 hours of administration of ascorbic acid. He was seen by a psychiatrist who noted a marked improvement in mood, though there was no documentation of any scoring for depression or anxiety. He was able to be discharged to sub-acute rehabilitation after four days. He was visited approximately seven days into his sub-acute rehabilitation stay and he was engaging in psychotherapy and physical therapy, improving social interactions with family, and eating regularly. A primary care visit 22 days after supplementation noted his motivation was better with improved mood and interactivity. He was not seen by a psychiatrist for follow-up. He was living with a cousin and his depressive symptoms improved. The exam at this clinic visit noted the resolution of abdominal ecchymosis. Follow-up with his primary care physician revealed a significant improvement in the patient's cutaneous symptoms, and he had markedly improved his self-care and outlook. He was well-groomed, interactive, and had made a plan with his cousin to improve his home life.

## Discussion

In the modern era of fortified diets, scurvy is rare in industrialized countries, except in patients with a history of alcohol or drug dependence with severely restricted diets devoid of fruits and vegetables. Institutionalized and socially isolated patients represent a population at increased risk of the disease [2, 8]. Healthy individuals with normal diets transiently hold approximately 1,500mg of total body VC with the highest concentrations found in the cutaneous tissues [1]. If not replenished with diet, this store depletes within one to three months and manifestations of scurvy begin to appear [3].

Vitamin C deficiency impairs collagen synthesis, which weakens blood vessels and makes them more prone to rupture, leading to ecchymosis and other bleeding disorders. Collagen is an important structural protein in the walls of blood vessels that provides tensile strength and resistance to deformation. Inadequate vitamin C levels lead to impaired collagen synthesis resulting in weakened vessel walls that are prone to rupture, bleeding, ecchymosis, and other bleeding disorders such as petechiae and purpura [9,10].

Several studies have investigated the relationship between vitamin C deficiency and bleeding disorders. A study conducted on guinea pigs demonstrated that vitamin C deficiency resulted in a reduction of collagen content in their vessel walls, leading to hemorrhages and subcutaneous bleeding [11]. In another study, vitamin C supplementation was shown to improve platelet function and reduce the risk of bleeding in patients with uremia [12]. A systematic review of studies on vitamin C and bleeding disorders concluded that vitamin C deficiency is a significant risk factor for bleeding and that vitamin C supplementation can improve bleeding time and reduce the incidence of bleeding. [13].

Clinical manifestations resulting from the friability of tissues and vasculature include gingival hyperplasia and hemorrhage, “corkscrew” body hair with perifollicular hyperkeratosis and perifollicular petechial hemorrhage, and ecchymosis with minor trauma [14].

The mechanism behind the formation of corkscrew hair in scurvy is not well understood. However, it has been suggested that vitamin C plays a role in the regulation of hair follicle development and maintenance, including the production of collagen, a major component of the hair shaft. Corkscrew hair is a characteristic feature of scurvy, and it is caused by the weakening of the hair shaft due to vitamin C deficiency, which leads to abnormal keratinization of the hair follicles [15]. The hair becomes thin and brittle, and the hair shafts lose their normal elliptical shape and become twisted, resembling a corkscrew. Vitamin C is required for the hydroxylation of proline and lysine residues in collagen, which stabilizes the triple helix structure of collagen molecules and promotes the formation of strong and resilient tissues [16]. It is worth noting that corkscrew hair is not specific to scurvy and can also be seen in other conditions that affect the hair shaft, such as trichothiodystrophy, a rare genetic disorder. [17].

Prolonged cases of vitamin C deficiency may eventually progress to the notorious breakdown of previously well-healed scars. Additional systemic effects of scurvy are seen in advanced cases such as edema, diarrhea, xerosis, and anemia as well as neuropsychiatric prodromal symptoms early in the disease process including lethargy, classically described as lassitude, and weakness that can be mistaken for depression. Advanced neurologic effects seen in later diseases include peripheral neuropathy and seizures [3]. Anemia is frequently associated with scurvy and may be the result of reduced iron absorption in the absence of dietary VC but also in hemolysis in advanced cases [2, 3].

Scurvy is diagnosed clinically and is supported by confirming low serum levels of ascorbic acid. A preferred alternative to plasma vitamin C levels is the measurement of leukocyte vitamin C concentration as it is a more accurate reflection of long-term intake of vitamin C and tissue stores; however, this test is less commonly available [18]. Treatment of scurvy is by oral or IV vitamin C supplementation, which can show symptomatic improvement over the course of approximately seven days, though initial clinical improvements can be seen in as little as 24 hours [2]. A wide range of replacement doses has been shown to be efficacious. Therefore, replacement should be individualized to the patient and can range from 300mg to 1000mg daily for one month [2, 14, 19].

Scurvy remains an important cause of mortality in the global context despite the perceived ease of prevention and cure. The World Food Programme (WFP) released an update to the Global Report on Food Crises to draw attention to a sharp increase in economic and political instability caused by the Covid-19 pandemic that has exacerbated ongoing climate-related food crises of which scurvy remains an issue [20].

**Figure 4:**
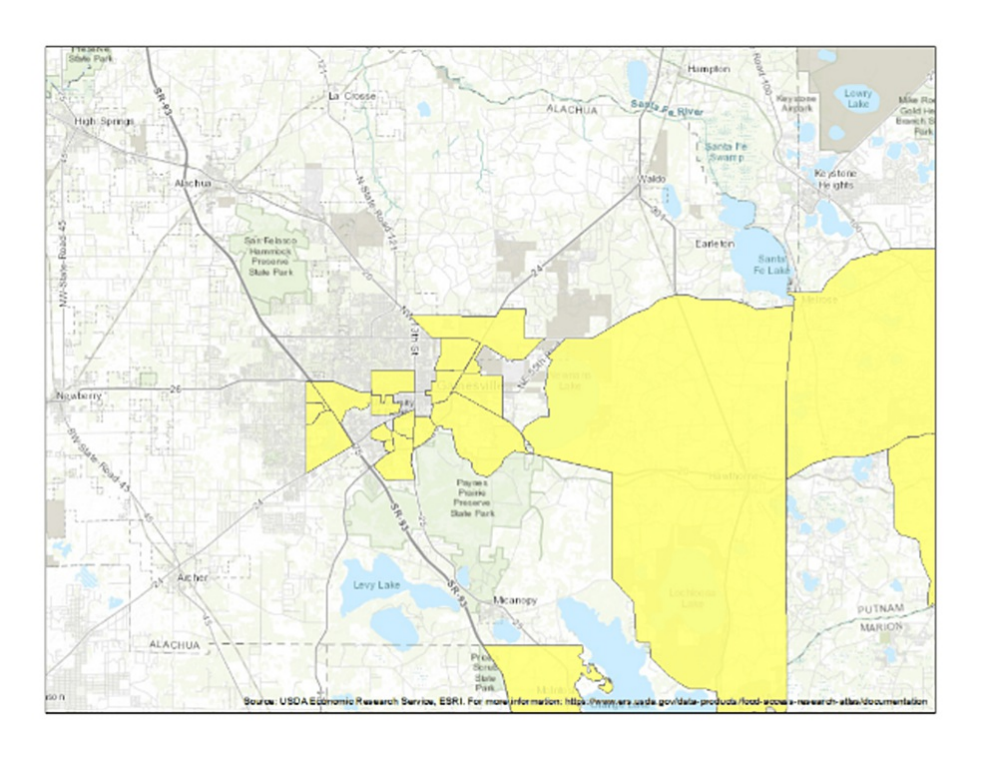
Community tracts in central Florida, USA with households USDA Community tracts in central Florida, USA with households USDA classified as low income, without access to transportation, and greater than ½ mile from grocery market

Food insecurity and food deserts are increasingly recognized in the North American context. For example, our hospital service area is located in the central Florida region where approximately 140,398 inhabitants have a median household income of $40,937 and 28.5% live at or below the federal poverty threshold of $13,788 in 2021 [21]. For perspective on the proximity of food insecurity, geospatial analysis of our medical service area in central Florida with US Department of Agriculture population data showing areas classified as low-income without access to public or private transportation and living greater than ½ mile from the nearest grocery market (Figure 4). 

**Figure 5:**
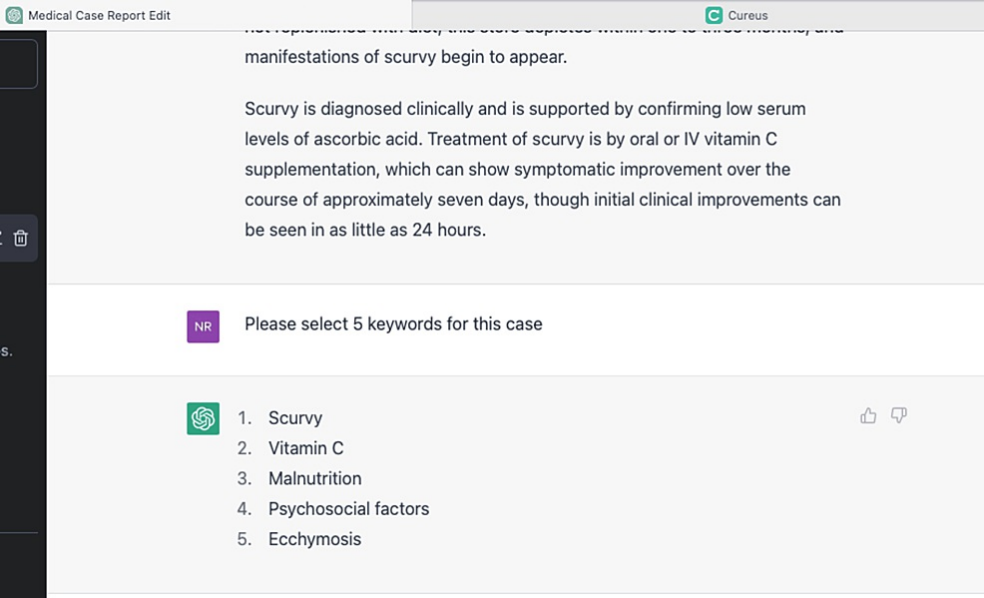
ChatGPT helps to find keywords

ChatGPT was used to assist with this case report. The background, case, discussion, and conclusions represent the original work of the authors, ChatGPT was used to find keywords Figure 5.

**Figure 6:**
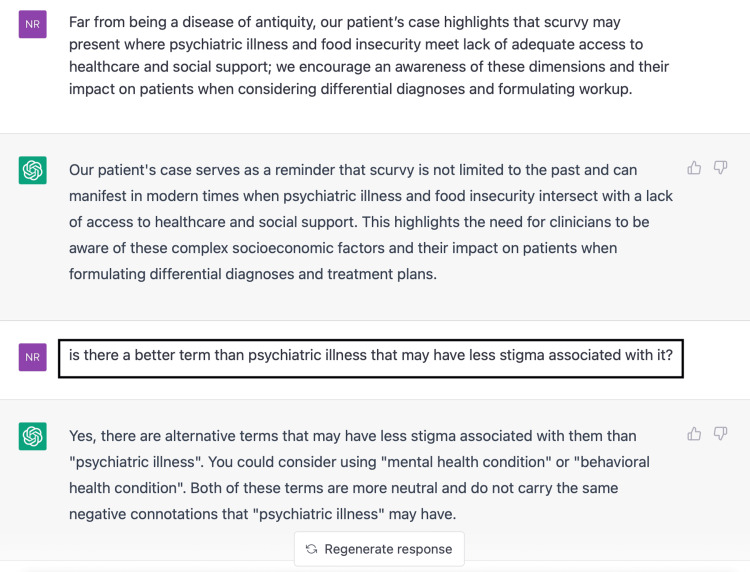
ChatGPT helps find a better phrase when prompted

We did not use it for any writing of this article other than to help with the writing style. ChatGPT was not used to make any diagnosis or to develop conclusions Figure 6.

The limitations of open artificial intelligence are still significant so all medical case reports will need authors to guide and confirm what is written. ChatGPT uses a database that is several years old and so that poses a limitation. Another limitation and risk would be the introduction of any sensitive patient information or sensitive scientific information. In fact, many companies have asked employees not to enter any sensitive or proprietary information into ChatGPT. 

## Conclusions

It is important to know about and recognize scurvy as it is still seen in modern times. Indeed, epidemic scurvy is still reported in some areas of the world. Specifically, intensifying regional conflicts have caused catastrophic famine conditions, wherein an extreme lack of food is leading to widespread starvation and death for an estimated 8 million people and an additional 20 million in nearing catastrophic famine conditions. Paradoxically, these famine conditions increasingly occur amongst regions of the world with the highest-rated soil fertility and are an important indicator of the transcendent sociopolitical dimensions of ancient and modern scurvy.

Our patient presented to a North American hospital in an urban setting with acute, though non-specific complaints from which initial workup did not clarify a diagnosis until the suspicion of scurvy arose after exploring a more thorough psychosocial history. Our patient's case serves as a reminder that scurvy is not limited to the past and can manifest in modern times when mental health conditions and food insecurity intersect.
